# Transcriptional level of inflammation markers associates with short-term brain structural changes in first-episode schizophrenia

**DOI:** 10.1186/s12916-023-02963-y

**Published:** 2023-07-10

**Authors:** Long-Biao Cui, Xian-Yang Wang, Yu-Fei Fu, Xiao-Fan Liu, Yongbin Wei, Shu-Wan Zhao, Yue-Wen Gu, Jing-Wen Fan, Wen-Jun Wu, Hengfen Gong, Bochao Danae Lin, Hong Yin, Fanglin Guan, Xiao Chang

**Affiliations:** 1 Department of Radiology, The First Affiliated Hospital of Xi’an Jiaotong University, Xi’an, China; 2 Shaanxi Provincial Key Laboratory of Clinic Genetics, Fourth Military Medical University, Xi’an, China; 3 Schizophrenia Imaging Lab, Fourth Military Medical University, Xi’an, China; 4 Department of Radiology, The Second Medical Center, Chinese PLA General Hospital, Beijing, China; 5 School of Artificial Intelligence, Beijing University of Posts and Telecommunications, Beijing, China; 6 Department of Psychiatry, Xijing Hospital, Fourth Military Medical University, Xi’an, China; 7 School of Medicine, Shanghai Pudong New Area Mental Health Center, Tongji University, Shanghai, China; 8 Department of Psychiatry and Neuropsychology, School for Mental Health and Neuroscience, Maastricht University Medical Centre, Maastricht, The Netherlands; 9 Department of Radiology, Xi’an People’s Hospital (Xi’an Fourth Hospital), Xi’an, China; 10 Department of Forensic Psychiatry, School of Medicine & Forensics, Xi’an Jiaotong University Health Science Center, Xi’an, China; 11 Institute of Science and Technology for Brain-Inspired Intelligence, Fudan University, Shanghai, China; 12 Key Laboratory of Computational Neuroscience and Brain-Inspired Intelligence, Ministry of Education, Fudan University, Shanghai, China; 13 MOE Frontiers Center for Brain Science, Fudan University, Shanghai, China

**Keywords:** Schizophrenia, Brain structure, Longitudinal alterations, Inflammation, Transcriptome

## Abstract

**Background:**

Inflammation has been implicated in the pathology of schizophrenia and may cause neuronal cell death and dendrite loss. Neuroimaging studies have highlighted longitudinal brain structural changes in patients with schizophrenia, yet it is unclear whether this is related to inflammation. We aim to address this question, by relating brain structural changes with the transcriptional profile of inflammation markers in the early stage of schizophrenia.

**Methods:**

Thirty-eight patients with first-episode schizophrenia and 51 healthy controls were included. High-resolution T1-weighted magnetic resonance imaging (MRI) and clinical assessments were performed at baseline and 2 ~ 6 months follow-up for all subjects. Changes in the brain structure were analyzed using surface-based morphological analysis and correlated with the expression of immune cells-related gene sets of interest reported by previous reviews. Transcriptional data were retrieved from the Allen Human Brain Atlas. Furthermore, we examined the brain structural changes and peripheral inflammation markers in association with behavioral symptoms and cognitive functioning in patients.

**Results:**

Patients exhibited accelerated cortical thickness decrease in the left frontal cortices, less decrease or an increase in the superior parietal lobule and right lateral occipital lobe, and increased volume in the bilateral pallidum, compared with controls. Changes in cortical thickness correlated with the transcriptional level of monocyte across cortical regions in patients (*r* = 0.54, *p* < 0.01), but not in controls (*r* =  − 0.05, *p* = 0.76). In addition, cortical thickness change in the left superior parietal lobule positively correlated with changes in digital span-backward test scores in patients.

**Conclusions:**

Patients with schizophrenia exhibit regional-specific cortical thickness changes in the prefrontal and parietooccipital cortices, which is related to their cognitive impairment. Inflammation may be an important factor contributing to cortical thinning in first-episode schizophrenia. Our findings suggest that the immunity-brain-behavior association may play a crucial role in the pathogenesis of schizophrenia.

**Supplementary Information:**

The online version contains supplementary material available at 10.1186/s12916-023-02963-y.

## Background

Schizophrenia is a severe mental disorder ranked among the top 20 causes of disability worldwide [1]. The prolonged illness course and impaired cognitive functioning are the main reasons contributing to the disease burden [2, 3]. Yet, the pathological mechanism of schizophrenia remains to be unclear. Imaging genetics study provides an opportunity to quantify disease-related neuroanatomical deviations and to elucidate the potential biological mechanism underlying these changes. In recent years, imaging genetics analysis has been increasingly applied to schizophrenia to reveal its potential pathological pathway [4, 5].

The suggestion that immune dysfunction may contribute to the pathophysiology of schizophrenia has a long history [6]. The vulnerability-stress-inflammation model [7] proposes that genetic risks and early life exposures, such as stress or infection, may promote a chronic pro-inflammatory state and subsequently lead to neurotransmission disturbances and brain structural changes that are relevant for schizophrenia [7, 8]. Supporting this hypothesis, therapeutic studies indicated a beneficial effect of using anti-inflammatory medications as an add-on treatment in the early stage of schizophrenia [7]. Studies of inflammation indicated that genetically predicted IL-6 was associated with brain volume in the middle temporal gyrus and may potentially be involved in the pathology of schizophrenia [9]. Likewise, studies also found overlapped genetic loci of schizophrenia and cortical morphometry to be enriched in immunologic gene sets [4]. However, few longitudinal studies have examined whether treatment-associated brain structural changes are related to neuroinflammation or gene expression of inflammatory markers.

Studies have indicated that progressive brain structural changes have been shown in the general population and patients with schizophrenia [10, 11]. In schizophrenia, previous studies found subcortical enlargement and cortical thinning in widespread brain regions [11, 12]. The frontal and temporal cortices were the most affected regions with excessive cortical thinning in patients than controls [11, 13]. Several large-scale, longitudinal studies have been conducted on schizophrenia [14], and a few studies focused on the early stage of illness when the majority of patients were medication-naïve and experienced substantial symptom alleviation [15, 16]. However, most studies did not recruit controls for follow-up, and it is difficult to distinguish the effects of disease progression and natural aging.

Considering that surface area and cortical thickness reflect different aspects of neural development processes [17] and may be differently affected in schizophrenia, we separately analyzed cortical thickness, surface area, and subcortical volume using surface-based morphometry (SBM). The aim of this study is threefold: (1) to determine the early-stage brain structural changes after a short period of treatment in patients with schizophrenia, (2) to investigate factors (inflammation and antipsychotic treatment) that may contribute to brain structural changes by correlating structural changes with transcription level of gene sets of interest, and (3) to evaluate the association of brain structural changes with changes in symptoms and cognitive performances in patients.

## Methods

### Participants

In this study, 38 first-episode patients with schizophrenia were recruited during their hospitalization in the Department of Psychiatry, Xijing Hospital, between May 2015 and October 2017 [18] (Table 1). The diagnosis was based on the Diagnostic and Statistical Manual of Mental Disorders, Fifth Edition [19]. Patients were included if this was the first time they were in the hospital or sought outpatient clinical help for their mental disorders. Additional inclusion criteria for patients require no more than 2 weeks of cumulative exposure to antipsychotics [20]. The exclusion criteria were listed in the Additional file 1: Supplementary Methods [20, 21]. All patients received second-generation antipsychotic medications determined by the clinicians during the study period, and 36 out of 38 patients received combined rTMS treatment during hospitalization (Additional file 1: Supplementary Methods) [22]. After a period of treatment (mean treatment duration: 148.29 ± 56.20 days), patients received a follow-up MRI scanning and assessment of clinical symptoms and cognitive functions.

**Table 1:** Demographic and clinical profile of 38 first-episode patients with schizophrenia and 51 healthy controls

	Controls	Patients	Between-group comparisons (*p*, *t* or *χ*^2^)^a^	
Age (years)	21.14 ± 4.03	23.03 ± 6.46	0.09	*t* = − 1.69
Sex (male/female)	33/18	21/17	0.37	*χ*^2^ = 0.81
Education (years)	13.89 ± 2.65	12.26 ± 2.97	0.01	*t* = 2.72
Follow-up interval (days)	148.29 ± 56.20	131.51 ± 4.46	0.04	*t* = 2.13
Illness duration (years)^b^	/	0.46 (0.17, 1)		*/*
Stay in hospital (days)	/	18.45 ± 8.25		*/*
Antipsychotics (olanzapine equivalent, mg/day)^b,c^	/	9.84 (6.78, 14.94)		*/*

^a^Group comparison was conducted using two-sample *t*-test. Nominal variables (sex) were compared with chi-square statistics

^b^Illness duration and dose of antipsychotic medication did not follow a normal distribution; therefore, the median (25th, 75th percentile) values were reported

^c^Antipsychotic dose was converted to a defined daily dose (https://doi.org/10.1093/schbul/sbv167)

Fifty-one healthy controls were recruited from the local community through advertisement and social media, with gender- and age-matched to the patient group (Table 1). The healthy control group received MRI scanning and cognitive assessment at recruitment and follow-up periods (131.51 ± 4.46 days). Written informed consent was signed by all subjects or parents of subjects under 18 years of age, after understanding this study in detail.

### Clinical assessment, cognitive tests, and laboratory tests

Symptom severity of the enrolled patients was evaluated using the Positive and Negative Syndrome Scale (PANSS) at baseline and follow-up time points [23]. PANSS subscale scores were calculated including positive symptoms, negative symptoms, and general psychopathology. We used ΔPANSS to indicate the degree of symptom relief in patients after treatment (T_1_) and before treatment (T_0_): ∆PANSS = (PANSS_T_1_ − PANSS_T_0_)/(PANSS_T_0_ − 30). The Wechsler Adult Intelligence Scale (WAIS), revised in China [24], was applied to patients and controls to evaluate their cognitive functioning. We performed the WAIS digital span (forward and backward) test and digital symbol coding test on all participants. In addition to the MRI scans, we also collected routine laboratory records of blood samples from the clinical database. Specifically, 2 ml of intravenous blood was collected from the upper forearm in an EDTA vacuum tube under fasting condition after admission, and the samples were measured using a Sysmex XS Automatic Hematology Analyzer. The data on leukocyte count and percentage were extracted from baseline laboratory tests to analyze the inflammatory indicators.

### MRI acquisition and preprocessing

High-resolution T1-weighted MRI was acquired using a GE Discovery MR750 3.0 T scanner located in the Department of Radiology, and all subjects underwent T2WI scans to rule out organic diseases. Participants showing gross artifacts and/or excessive head motion were excluded, following protocols established previously [21]. The MRI acquisition was accomplished at baseline and follow-up, at the same time as the clinical assessment. The scanning parameters were repetition time 8.2 ms, echo time 3.2 ms, flip angle 12°, field of view 256 × 256 mm, matrix 256 × 256, slice thickness 1 mm, and sagittal slices 196. The image preprocessing followed the analysis process of FreeSurfer (version 6.0.0, https://surfer.nmr.mgh.harvard.edu) and was performed on the Linux operating system. The brief preprocessing steps included correcting the uniformity of the bias field; removing the skull; segmenting the brain tissue into gray matter, white matter, and cerebrospinal fluid; cortical surface reconstruction; registration of individual space to the standard space by nonlinear transformation; the registered image was segmented into 68 cortices based on Desikan-Killiany atlas and 14 subcortical regions. For each subject, 150 regional structural measurements were obtained, including 68 regional cortical thicknesses, 68 surface areas, and 14 subcortical volumes.

### Linear mixed-effect model on brain structural changes

The statistical analysis was performed on MATLAB (R2020b) platform. A linear mixed-effect model was applied to simultaneously test the group differences (group), time point (time), and their interaction effect on global and regional morphological measurements (*Y*). Age and sex were included as covariates. When comparing regional measurements, we additionally included global measurements to adjust for global differences between individuals. Each subject was given a subject ID (SubID) which was included as the random factor. Multiple testing effect was corrected using false discovery rate (FDR) correction with *q* < 0.05 for regional measurement comparisons.

### Brain transcriptional data

Brain transcriptional data were obtained from the Allen Human Brain Atlas (AHBA) [25] (http://human.brain-map.org/), including 20,734 genes across the brain from six healthy human donors (five males, age range of 24 ~ 57). For each tissue sample, gene expression data represented by 58,692 probes were quantified, normalized, and averaged across probes following a previous study [26]. We used gene expression data from the left hemisphere as they are available from all donors. Tissue samples were mapped to the Desikan-Killiany atlas [27] based on Euclidean distance to the nearest voxel in the MNI 152 template [26]. For each gene, the expression level was averaged across the six donors and normalized to *z* score across all cortical regions, resulting in a normalized gene expression matrix of size 34 × 20,734 (region × genes) for further analysis. Detailed processing steps are described in Additional file 1: Supplementary Methods [25–28].

To identify inflammation-related genes, we selected genes from a previous systematic review on leukocytes and subtypes (monocyte, lymphocyte, neutrophil, eosinophil, and basophil) [29]. To reduce potential bias in gene set selection, we further verified our results using inflammation-related genes reported in another review [30]. Genes related to antipsychotic treatment response were selected from a genome-wide association study (GWAS) on antipsychotic treatment in schizophrenia [31]. Included genes are listed in Additional file 1: Table S1.

### Association between brain structural changes and peripheral inflammation markers

We next examined the associations between brain structural changes and baseline peripheral inflammation markers in patients. Inflammation markers were quantified including leukocyte count and percentage of leukocyte subtypes (monocyte, lymphocyte, neutrophil, eosinophil, and basophil) (Table 2). As some inflammation markers do not follow a normal distribution (Additional file 1: Table S2), Spearman’s rank correlation was performed between inflammation markers and structural changes in the brain regions that showed a significant group main effect or group by time interaction effect. We further examined the associations between inflammation markers and subcortical volume changes, as some previous studies reported an association between subcortical volume and peripheral inflammatory markers [32], but not consistently [33].

**Table 2:** Leukocyte count and leukocyte subtypes (percentage) in patients

	Leukocyte (10^9^/L)	Monocyte (%)	Lymphocyte (%)	Neutrophils (%)	Eosinophils (%)	Basophils (%)
Median	5.645	6.6	36.2	54.6	1.7	0.2
25% percentile	4.79	5.5	29.8	46.9	1.1	0.2
75% percentile	6.58	7.2	44.1	63	2.6	0.4

### Association between brain structural changes and transcriptional profiles

We next investigated whether brain structural changes were associated with gene expression patterns related to inflammation and antipsychotic response (Additional file 1: Table S1). For each set of genes of interest, normalized gene expression was averaged across samples. The normalized gene expression maps were cross-correlated with group-averaged cortical thickness and surface area changes in patients. The same correlation analysis was also performed in controls to examine whether the effects were unique to patients.

As recent studies pointed out that spatial autocorrelation of brain maps results in false-positive effects [34], we verified our correlation results between brain and transcriptional maps using generative null models proposed by Burt et al. [35]. Specifically, 5000 surrogate maps preserving spatial autocorrelation of brain structural changes in patients were generated using the BrainSMASH toolkit [35]. The null distribution of correlations between gene expression and surrogate brain maps was calculated and compared with our empirical results. The two-sided *p* value was determined as the proportion of random permutations that exceeded the empirical correlation coefficient between gene expression and brain maps.

### Brain structural changes in relation to clinical profile and cognitive functioning

To determine the behavioral relevance of brain structural changes in patients, we performed Pearson correlation between brain structural changes in regions showing a significant group or group by time interaction effect and clinical factors: PANSS subscale score changes and olanzapine equivalent antipsychotic dosage. We also examined the correlations between brain structural changes and WAIS digital symbol coding and digital span (forward and backward) score changes in both patients and controls. Multiple testing (11 regions × 7 behavioral items) was corrected using the FDR at *q* < 0.05.

### Peripheral inflammation markers in relation to clinical profile and cognitive functioning

Spearman’s rank correlation was performed between inflammation markers (leukocyte and leukocyte subtypes) and patients’ behavioral scores (PANSS and WAIS subscales) at baseline and follow-up.

## Results

### Demographic characteristics and clinical outcomes

Demographic and clinical information of all participants were shown in Table 1. Controls were matched with patients on age (*p* = 0.09) and sex (*p* = 0.37), and controls had a higher education level (*p* = 0.01) and shorter follow-up interval (*p* = 0.04) than patients. The patient’s symptom severity at baseline and follow-up period was illustrated in Table 3. The majority of patients (33 out of 38) had an improvement in their symptoms, with PANSS total score reduced by more than 30% (Additional file 1: Fig. S1). On cognitive assessments, patients showed significantly lower scores on WAIS digital symbol coding and digital span (forward and backward) tests compared to controls (all *p* < 0.001). Patients did not show significant improvements in WAIS scores at follow-up. Information on patients who were medicated before admission is listed in Additional file 1: Table S3.

**Table 3:** PANSS score and cognitive functions before and after treatments

	Baseline	Follow-up	Within-group comparisons (*p*, *t*)		Between-group comparisons (*p*, *t*)	
PANSS						
Total score	89.11 ± 14.81	51.76 ± 13.14	< 0.001	12.61	*/*	
Positive	22.90 ± 4.96	10.32 ± 4.01	< 0.001	12.57	*/*	
Negative	21.53 ± 6.40	14.53 ± 4.94	< 0.001	6.69	*/*	
General psychopathology	44.68 ± 8.89	26.92 ± 6.57	< 0.001	10.70	*/*	
Digital symbol coding test						
Controls	69.71 ± 9.19	78.69 ± 8.22	< 0.001	7.36	< 0.001	10.48
Patients	45.97 ± 12.19	48.59 ± 13.03	0.18	1.38		
Digital span forward test						
Controls	9.88 ± 1.03	9.43 ± 1.10	0.03	− 2.29	< 0.001	4.29
Patients	8.68 ± 1.60	8.91 ± 1.54	0.25	1.16		
Digital span backward test						
Controls	7.45 ± 1.63	7.84 ± 1.27	0.05	2.00	< 0.001	7.19
Patients	4.82 ± 1.81	4.51 ± 1.48	0.30	− 1.06		

### Group comparisons of brain structural changes

#### Global effects

**Fig. 1:**
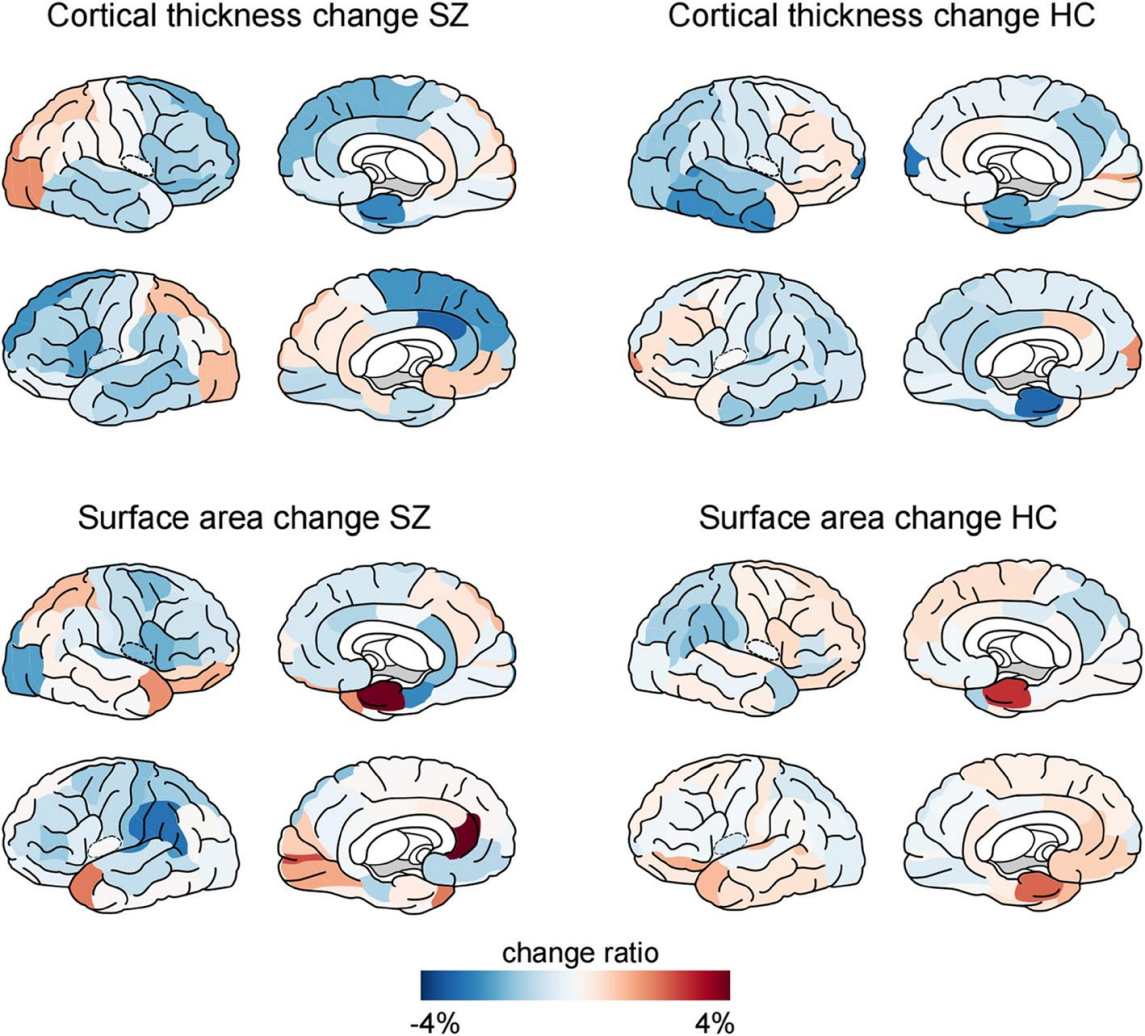
Percentage of cortical thickness (upper panel) and surface area (lower panel) changes in patients with schizophrenia (left) and healthy controls (HC). Blue color indicates a decreased value at follow-up compared to baseline measurements, whereas red color indicates an increased value

In both patients and controls, brain structural measurements decreased at the follow-up time point compared to baseline (Table 4). Global volume, cortical thickness, and surface area on average decreased by 1.08%, 0.87%, and 0.65% in patients and 0.97%, 0.76%, and 0.24% in controls, respectively (Fig. 1). Linear mixed-effect model showed a significant main effect of time on global volume (*t* =  − 2.34, *p* = 0.02) and cortical thickness (*t* =  − 2.36, *p* = 0.02), but not for surface area (*t* =  − 0.52, *p* = 0.61). No significant group main effect or group by time interaction effect was found in global measurements.

**Table 4:** Global structural measurements and change ratio in patients and controls

	Baseline	Follow-up	Change ratio
Global GMV (mm^3^)			
Controls	5.90 × 10^5^	5.85 × 10^5^	− 0.97%
Patients	5.72 × 10^5^	5.65 × 10^5^	− 1.08%
Global cortical thickness (mm)			
Controls	177.44	176.15	− 0.76%
Patients	175.69	174.14	− 0.87%
Global surface area (mm^2^)			
Controls	1.85 × 10^5^	1.85 × 10^5^	− 0.24%
Patients	1.81 × 10^5^	1.80 × 10^5^	− 0.65%

#### Regional effects

**Fig. 2:**
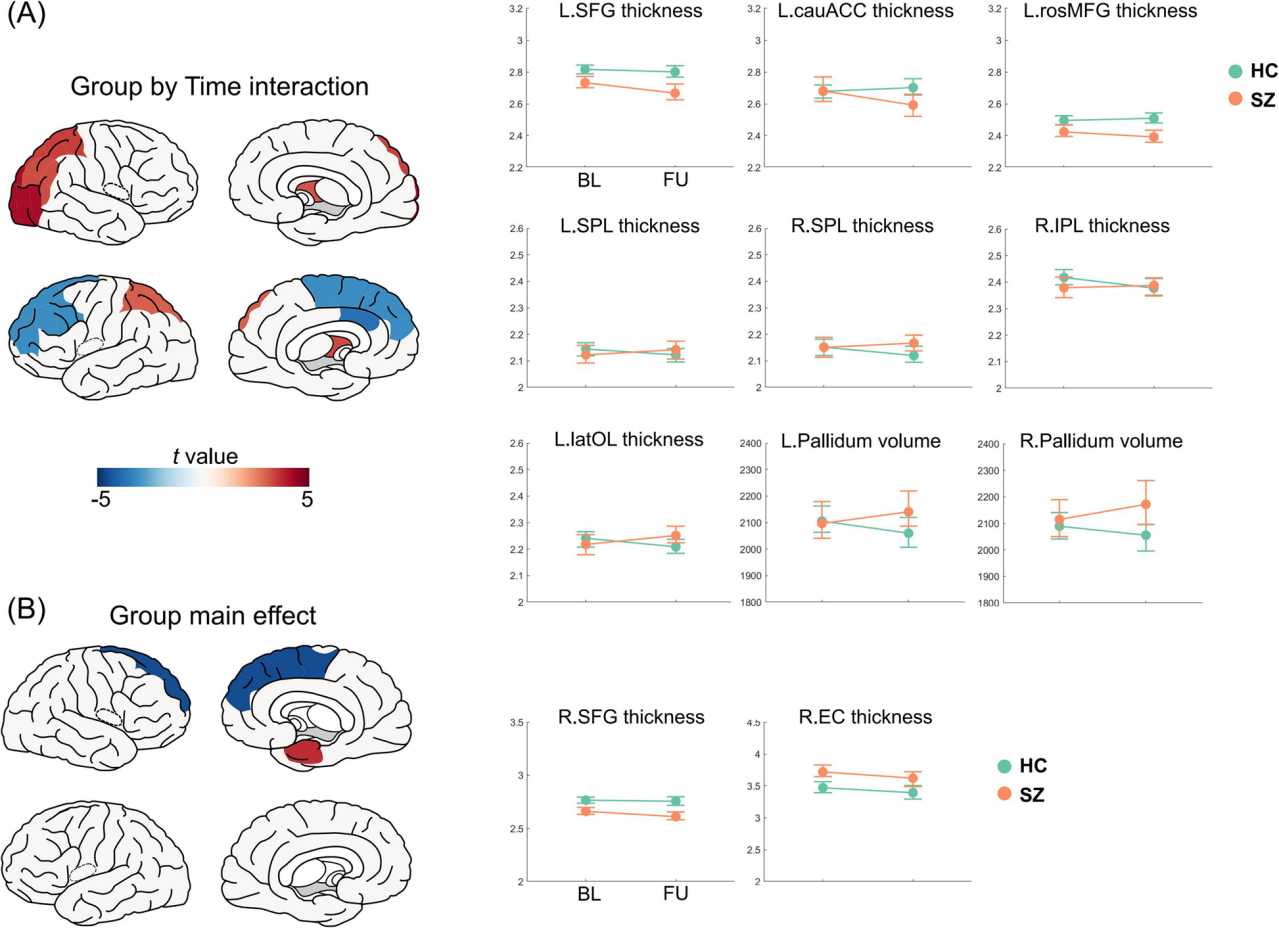
Significant group by time interaction effect (**A**) and group effect (**B**) on brain structural measurements. *T*-statistics from linear mix-effects model was plotted on the left, with blue color indicating lower values or accelerated decrease in patients, while red indicates the opposite. For significant regions, cortical thickness and volume in patients (SZ) and controls (HC) at baseline (BL) and follow-up (FU) were plotted on the right.  Abbreviations: L: left, R: right, SFG: superior frontal gyrus, cauACC: caudal anterior cingulate cortex, rosMFG, rostral middle frontal gyrus, SPL: superior parietal lobule, IPL: inferior parietal lobule, latOL: lateral occipital lobe, EC: entorhinal cortex

For regional structural changes, we analyzed the group by time interaction effects and group main effects on 150 regional measurements (14 subcortical volumes, 68 cortical thicknesses, and 68 surface areas). For group by time interaction effects, several regional cortical thicknesses showed a significant result, including the left superior and middle frontal gyri, left caudal anterior cingulate gyrus, bilateral superior parietal lobules, right inferior parietal lobule, right lateral occipital lobe, and bilateral pallidum. For significant group main effects, the right superior frontal gyrus showed significantly lower cortical thickness whereas the right entorhinal cortex showed higher cortical thickness in patients than in controls (Fig. 2 and Table 5). No significant group or group by time interaction effects were found for surface area comparison. We conducted a sub-group analysis of patients who were medicated at baseline, which showed similar results (Additional file 1: Table S4).

**Table 5:** Significant group by time interaction and group main effects on regional measurements (FDR significant results in bold font)

	Group		Time		Group × time	
	*t* value	*p* value	*t* value	*p* value	*t* value	*p* value
Subcortical regions						
Right pallidum	1.00	0.32	− 1.10	0.27	**3.11**	**< 0.01**
Left pallidum	0.03	0.98	− 1.77	0.08	**3.28**	**< 0.01**
Regional cortical thickness						
Right entorhinal cortex	**3.66**	**< 0.01**	− 1.18	0.24	− 0.29	0.77
Right superior frontal gyrus	**− 4.55**	**< 0.01**	0.85	0.40	− 2.46	0.01
Left rostral middle frontal gyrus	− 2.61	0.01	3.72	< 0.01	**− 3.11**	**< 0.01**
Left caudal anterior cingulate gyrus	0.53	0.60	1.68	0.10	**− 3.80**	**< 0.01**
Left superior frontal gyrus	− 3.09	< 0.01	0.41	0.68	**− 3.10**	**< 0.01**
Left superior parietal lobule	− 0.55	0.58	− 0.61	0.54	**3.01**	**< 0.01**
Right inferior parietal lobule	− 0.94	0.35	− 1.88	0.06	**3.23**	**< 0.01**
Right lateral occipital lobe	− 0.57	0.57	− 1.47	0.14	**4.16**	**< 0.01**
Right superior parietal lobule	0.47	0.64	− 1.40	0.16	**3.43**	**< 0.01**

### Association between brain structural changes and peripheral inflammation markers

We first tested whether the brain regions that showed a significant group main effect or group by time interaction effect would correlate with patients’ peripheral inflammation levels. Next, we performed an exploratory analysis in the subcortical regions. We did not observe a significant association between structural changes and inflammation markers.

### Association between brain structural changes and transcriptional profiles

**Fig. 3:**
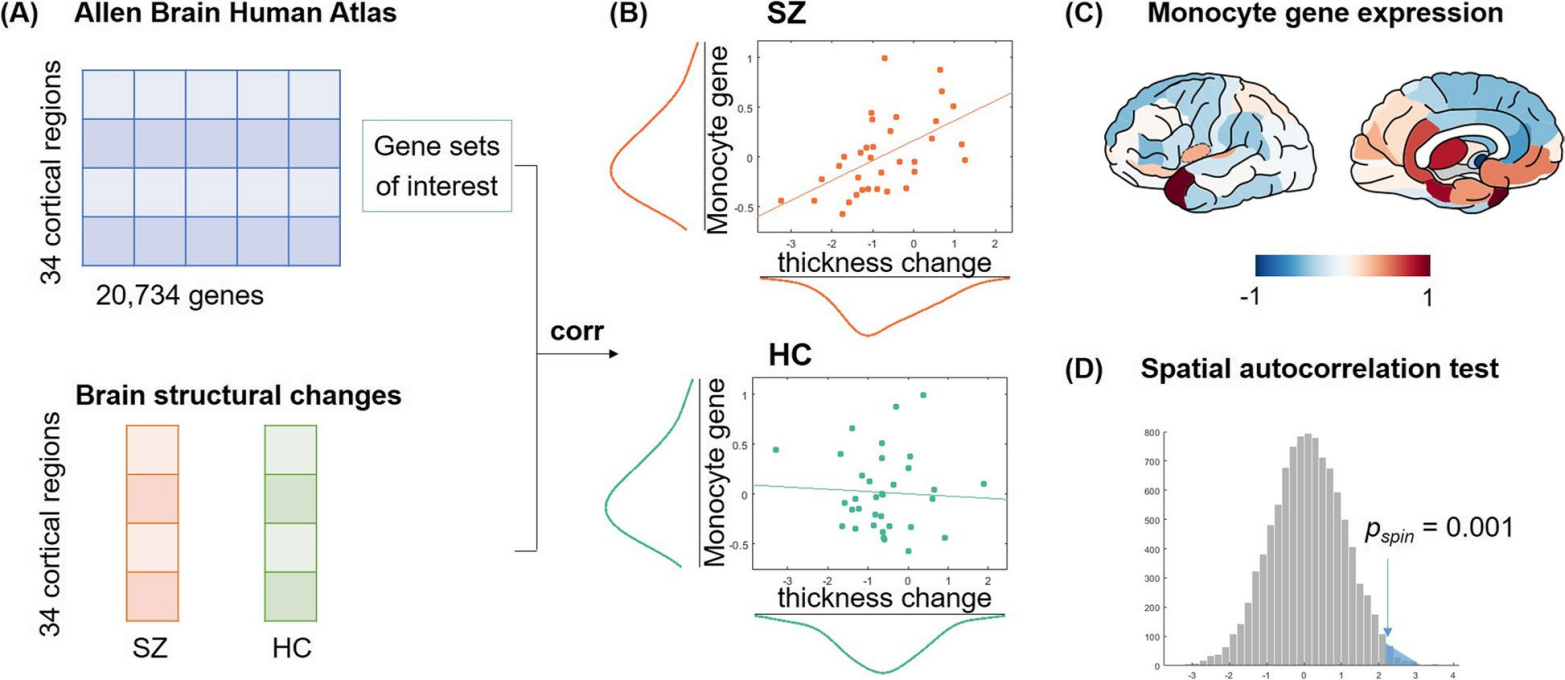
Association between brain structural changes and transcriptional profiles of inflammation and antipsychotic response. **A** Transcriptional data were retrieved from the Allen Human Brain Atlas (AHBA) [25] (http://human.brain-map.org/) and calculated for gene sets of interests [29–31] (Additional file 1: Table S1). Brain structural changes (cortical thickness and surface area) were calculated for patients with schizophrenia (SZ) and healthy controls (HC) as illustrated in Fig. 1. **B** A significant correlation was found between monocyte-related gene expression and cortical thickness changes in patients with schizophrenia (*r* = 0.54, *p* < 0.01) but not in healthy controls (*r* =  − 0.05, *p* = 0.76). **C** The normalized monocyte gene expression profile from a systematic review on leukocyte and subtypes [29]. **D** Association between monocyte gene expression and cortical thickness changes in patients is significantly higher than the null model generated by correlations between 5000 surrogate maps and monocyte gene expression profile (*p*spin = 0.001)

Next, we investigated whether brain structural changes correlated with the gene expression profile of inflammation and antipsychotic treatment (Additional file 1: Fig. S2). Our analysis revealed a positive correlation between cortical thickness changes and gene expression of monocyte [29] in patients (*r* = 0.54, *p* = 8.8 × 10^−4^, FDR corrected) but not in healthy controls (*r* =  − 0.05, *p* = 0.76) (Fig. 3 and Table 6). This result was replicated using inflammation genes from another study, which showed a positive correlation between cortical thickness changes and gene expression of monocyte in patients (*r* = 0.51, *p* = 0.002) [30]. We further tested whether this micro–macro association was driven by spatial autocorrelation of neuroimaging measurements. Our empirical results exceeded the null distribution of correlations between 5000 surrogate maps and monocyte gene expression (two-tail *p* = 0.001 for [29] and *p* = 0.006 for [30]). The correlation between cortical thickness changes and antipsychotic genes in patients is *r* = 0.37, *p* = 0.03, but did not survive FDR correction [31]. We also conducted a sub-group analysis of patients who were medicated at baseline, which showed a significant correlation with monocyte gene expression level (Additional file 1: Table S5).

**Table 6:** Correlation between structural changes and gene expression of antipsychotic treatment response and inflammation in patients (SZ) and controls (HC)

Neuroimaging	Study	Gene set of interests	SZ		HC	
			*r* value	*p* value	*r* value	*p* value
Cortical thickness change	Yu et al. (2018) [31]	Antipsychotics	0.159	0.370	− 0.327	0.059
	Nalls et al. (2011) [29]	Leukocyte	− 0.019	0.913	− 0.250	0.154
		Neutrophil	0.173	0.328	− 0.169	0.340
		Basophil	0.384	0.025	0.063	0.721
		Monocyte	**0.544**	**8.8 × 10**^**−4**^	− 0.054	0.762
		Lymphocytes	0.039	0.827	− 0.305	0.079
	Keller et al. (2014) [30]	Leukocyte	− 0.277	0.113	− 0.168	0.344
		Neutrophil	− 0.115	0.516	− 0.063	0.725
		Monocyte	**0.511**	**0.002**	− 0.188	0.288
Surface area change	Yu et al. (2018) [31]	Antipsychotics	0.367	0.033	0.163	0.356
	Nalls et al. (2011) [29]	Leukocyte	0.110	0.536	0.127	0.474
		Neutrophil	0.134	0.449	− 0.141	0.425
		Basophil	− 0.109	0.538	− 0.174	0.326
		Monocyte	0.007	0.970	0.240	0.172
		Lymphocytes	0.357	0.038	0.454	0.007
	Keller et al. (2014) [30]	Leukocyte	0.153	0.387	0.500	0.003
		Neutrophil	− 0.126	0.476	0.343	0.047
		Monocyte	0.095	0.594	0.173	0.328

### Brain structural changes in relation to clinical factors and cognitive functioning

**Fig. 4:**
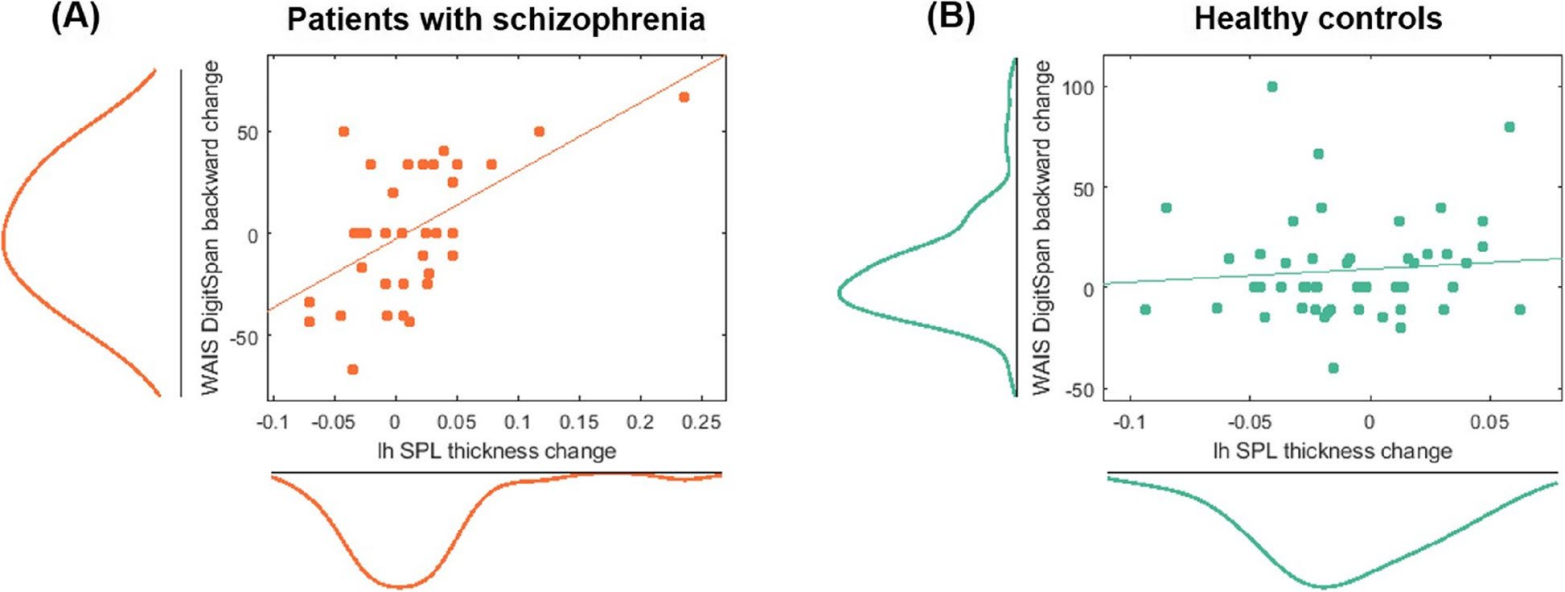
Correlation analysis between brain structure changes and cognitive score changes in patients (**A**) and controls (**B**). A significant association was found between the left superior parietal lobule (SPL) cortical thickness change and WAIS digital span (backward) change in patients (*r* = 0.57, *p* = 3.4 × 10^−4^) but not in controls (*r* = 0.09, *p* = 0.54)

For WAIS tests, we found that the left superior parietal lobule thickness change correlated with WAIS digital span (backward) change in patients (*r* = 0.57, *p* = 3.4 × 10^−4^) but not in controls (*r* = 0.09, *p* = 0.54) (Fig. 4). For PANSS score changes and medication dosage, we did not find any significant correlations with brain structural changes.

### Correlation between patients’ peripheral inflammation markers and clinical profile

We found an uncorrected association between monocyte percentage and PANSS positive score (Spearman’s *r* = 0.44, *p* = 0.006), as well as between monocyte percentage and digital span (backward) score (Spearman’s *r* = 0.38, *p* = 0.02) at baseline (Table 7). Yet, these correlations did not survive multiple corrections.

**Table 7:** Spearman’s correlation between patients’ inflammation markers and clinical scores

	Leukocyte		Neutrophils (%)		Lymphocyte (%)		Monocyte (%)		Eosinophils (%)		Basophils (%)	
	*p*	*r*	*p*	*r*	*p*	*r*	*p*	*r*	*p*	*r*	*p*	*r*
PANSS pos baseline	0.625	− 0.083	0.287	− 0.180	0.476	0.121	**0.006**	**0.443**	0.220	0.206	0.804	0.042
PANSS neg baseline	0.373	0.151	0.183	0.224	0.064	− 0.308	0.451	− 0.128	0.624	− 0.083	0.861	0.030
PANSS gen baseline	0.800	− 0.043	0.764	− 0.051	0.728	0.059	0.470	0.123	0.053	− 0.320	0.640	0.080
PANSS pos follow-up	0.765	0.053	0.721	0.064	0.904	− 0.021	0.557	− 0.104	0.396	− 0.150	0.098	− 0.288
PANSS neg follow-up	0.316	− 0.177	0.688	− 0.071	0.278	0.192	0.677	− 0.074	0.763	− 0.054	0.463	− 0.130
PANSS gen follow-up	0.308	0.180	0.555	0.105	0.299	− 0.184	0.160	− 0.246	0.660	− 0.078	0.349	− 0.166
DigitSymbol baseline	0.991	0.002	0.662	− 0.074	0.740	− 0.056	0.804	0.042	0.794	0.044	0.263	− 0.189
DigitSpan forward baseline	0.647	0.078	0.192	− 0.219	0.554	0.100	**0.019**	**0.384**	0.470	0.123	0.591	− 0.091
DigitSpan backward baseline	0.548	0.102	0.596	− 0.090	0.699	0.066	0.401	0.142	0.748	− 0.055	0.279	− 0.183
DigitSymbol follow-up	0.631	− 0.087	0.887	− 0.026	0.415	0.147	0.537	− 0.111	0.198	− 0.230	0.923	− 0.018
DigitSpan forward follow-up	0.789	− 0.048	0.421	0.145	0.273	− 0.196	0.226	− 0.217	0.346	− 0.169	0.968	− 0.007
DigitSpan backward follow-up	0.465	− 0.132	0.554	− 0.107	0.819	0.041	0.971	0.007	0.587	0.098	0.284	0.192

## Discussion

This longitudinal study examined the patterns of brain structural changes in patients with first-episode schizophrenia and investigated the contributing factors (inflammation and antipsychotic treatment) to brain structural changes. We found that patients had accelerated pallidum enlargement and frontal cortical thinning but preserved parietal and occipital cortical thickness as compared to controls. Among these structural changes, cortical thickness change in the left superior parietal lobule positively correlated with cognitive performance changes in patients. Notably, we observed a positive correlation between the gene expression level of monocyte and cortical thinning in patients. Taken together, our results identified early-stage brain structural changes and their correlation with the transcriptional profile of inflammatory markers, providing preliminary evidence of the underlying biological process that may contribute to longitudinal brain structural changes in schizophrenia.

Immunological abnormalities have long been reported to be involved in schizophrenia. In previous schizophrenia studies, higher peripheral monocyte count could be relevant to the pathophysiology [36] and has been considered a possible marker of microglia activation [37]. Inflammation in the brain involved both monocyte and microglia. Microglial cells and chemokines might recruit monocytes circulating in the peripheral blood into the brain [38], where neuroinflammation was exacerbated by these monocytes. The investigation of monocyte transcriptome demonstrated a shift in monocyte phenotype in different stages of schizophrenia [39]. In the current study, the transcriptional profile of monocyte was directly related to the longitudinal change of cortical thickness in patients, suggesting an association of monocyte gene expression with the effect of treatment and this disease itself on the brain.

However, we did not observe a significant association between structural changes and inflammation markers. Previous studies revealed elevated peripheral inflammatory levels in patients with schizophrenia and their correlation with increased basal ganglia volume [40]. The effect of peripheral inflammatory markers on brain structural changes was further supported by genetic studies implicating multiple biological processes such as neural development and synaptic transmission [9]. Leukocyte count, even within the normal range, was correlated with polygenic scores of schizophrenia [41]. Since the antipsychotic medication was related to the decrease of cytokines [42], and basal ganglia volume change was quite common after medication [43], one possible mechanism of antipsychotics in the treatment of psychosis could be mitigating neuroinflammation.

Although antipsychotics can alleviate symptoms of schizophrenia, some studies raised concern that they may contribute to additional cortical thinning [44]. Subsequent studies argued that cortical thinning after medication was not linked to clinical or cognitive deterioration [45] and might even improve patients’ prefrontal functional activity and cognitive control ability [13]. In a macaque monkey study, antipsychotic-induced volume reduction was related to reduced glial cell number, whereas the number of neurons remained unchanged [46]. In our study, we found the transcriptional profile of monocyte positively correlated with cortical thickness changes after treatment, indicating the important role of inflammation, especially monocyte, in cortical thickness changes in the early stage of schizophrenia.

Several other factors may contribute to structural changes in patients with schizophrenia, such as medication and neuromodulation treatment. Studies found that medication duration and dosage correlated with cortical thinning in patients [11, 45], with effects varied across different types of antipsychotics [14]. As a non-invasive adjuvant therapy, repetitive transcranial magnetic stimulation (rTMS) may prevent volume reduction or cortical thinning [47] and increase brain metabolism at the stimulation site [48]. The temporoparietal junction is a commonly applied target site.

We also analyzed the factors that might contribute to these structural changes, including medication, times of rTMS, and peripheral inflammatory markers. Although given the fact that treatment factors were able to cause structural changes, we did not find a significant correlation between brain changes and the medication dose, duration, or times of rTMS as former studies reported [11, 45]. Three reasons might contribute to this. First, it might be due to our lack of comparison to unmediated patients, since the disease course would also lead to cortical thinning even in prodromal high-risk individuals [49]. Thus, when calculating the effect of treatment, the effect was not linearly related to structural changes due to the interference of the disease course. Second, it could also be on account of the combined treatment of multiple antipsychotics for each patient, because some studies argued that different types of antipsychotics had varied influences on structural changes as well [14]. Lastly, different patients had different responses to antipsychotics, and our limited sample size might not suffice to prove the correlation with treatment factors.

Among the brain regions showing significant group or group by time interaction effects, we found a positive correlation between superior parietal lobule thickness change and WAIS digital span score change in patients, suggesting the cognitive relevance of this region. The superior parietal lobule belongs to the association cortex and is involved in high-order processes like cognition, information integration, and self-awareness [50]. Structural and functional abnormalities of the frontal-parietal network were frequently reported and have been related to cognition impairments in schizophrenia. Previous studies have reported that low-frequency rTMS could regulate functional connectivity within the frontal-parietal network and improve cognitive functioning [47]. Our study supported the role of the superior parietal lobule in working memory and suggested this potential region as a stimulation target site for cognitive remediation.

In the study, we found a regional-specific effect of accelerated frontal cortical thinning and pallidum enlargement as well as preserved parietal and occipital cortices that may be due to medication and rTMS, respectively. Our results were consistent with former cross-sectional studies [11–13, 43] and provided a longitudinal view in patients with first-episode schizophrenia who were unmedicated and responsive to treatments. Remarkably, consistent with previous meta-analysis [11] and longitudinal study [45], cortical thickness represented more prominent changes in regional analysis rather than surface area, which might reflect different mechanisms underlying these two measurements. It is assumed that the developmental trajectories of surface area were predominantly influenced by genetic factors [11, 17]. Meanwhile, cortical thickness can be affected by additional environmental or neurodegenerative factors (disease, inflammation, treatment, aging, etc.) even in adulthood [17]. Our study supported that cortical thickness might be more sensitive to treatment and inflammation effects.

Several limitations need to be considered when interpreting our results. First, transcriptomic data were not collected in this sample but retrieved from the external AHBA dataset, leading to false-positive effects in micro–macro association due to spatial autocorrelation. Using generative null models, the correlation results between brain and transcriptional maps were verified. Second, our findings of brain structural changes reflect mixed effects of treatment and disease progression on the brain, which cannot be easily separated apart. However, we tried to control for the time effect by including follow-up MRI measurements of healthy controls in linear mixed-effect models. Third, the sample size of this study was moderate. Future studies with a larger sample size or using a meta-analytic approach could further confirm our findings.

## Conclusions

In summary, this study found brain structural changes in patients with first-episode schizophrenia after short-term treatment. The cortical thickness change in patients was related to the gene expression level of inflammation, and cortical thickness in the superior parietal lobule correlated with ameliorated cognitive impairments in patients. These results provided empirical evidence of potential biological processes underlying brain structural changes and suggested possible treatment targets to improve cognitive function in patients. More importantly, identified immunity-brain-behavior associations would contribute to our understanding of the pathogenesis of schizophrenia.

## Supplementary Information

**Additional file 1: Supplementary Methods.** Exclusion criteria. rTMS treatment. Brain transcriptional data. **Table S1.** Genes of interest related to inflammation and antipsychotic treatment response. **Table S2.** Shapiro-Wilk and Shapiro-Francia normality tests for inflammation markers. **Table S3.** Antipsychotics received at baseline for patients with schizophrenia. **Table S4.** Significant group by time interaction, group main effects, and time main effects on regional measurements among patients who were medicated at baseline (FDR significant results in bold font). **Table S5.** Correlation between structural changes and gene expression of antipsychotic treatment response and inflammation among patients who were medicated at baseline. **Fig. S1.** Change on PANSS score at baseline (T_0_) and follow-up period (T_1_). **Fig. S2.** Transcriptional level of gene sets of interest.

## Data Availability

Data used in the present study can be accessed upon request from the corresponding authors. The code of the project is available at https://github.com/xchang007/SCZ_treatment/tree/main.

## Abbreviations

AHBA: Allen Human Brain Atlas
FDR: False discovery rate
GMV: Gray matter volume
GWAS: Genome-wide association study
rTMS: Repetitive transcranial magnetic stimulation
SBM: Surface-based morphometry

## References

[1] James SL, Abate D, Abate KH, Abay SM, Abbafati C, Abbasi N, et al. Global, regional, and national incidence, prevalence, and years lived with disability for 354 diseases and injuries for 195 countries and territories, 1990–2017: a systematic analysis for the Global Burden of Disease Study 2017. Lancet. 2018;392(10159):1789–858. 10.1016/S0140-6736(18)32279-7 PMC6227754 30496104

[2] Wu X-S, Yan T-C, Wang X-Y, Cao Y, Liu X-F, Fu Y-F (2021). Magnetic resonance imaging-guided and navigated individualized repetitive transcranial magnetic stimulation for cognitive impairment in schizophrenia. Neurosci Bull.

[3] Zhao Q, Li J, Xiao Y, Cao H, Wang X, Zhang W (2021). Distinct neuroanatomic subtypes in antipsychotic-treated patients with schizophrenia classified by the predefined classification in a never-treated sample. Psychoradiology.

[4] Cheng W, Frei O, van der Meer D, Wang Y, O’Connell KS, Chu Y (2021). Genetic association between schizophrenia and cortical brain surface area and thickness. JAMA Psychiat.

[5] Patel Y, Parker N, Shin J, Howard D, French L, Thomopoulos SI (2021). Virtual histology of cortical thickness and shared neurobiology in 6 psychiatric disorders. JAMA Psychiat.

[6] Buckley PF (2019). Neuroinflammation and schizophrenia. Curr Psychiatry Rep.

[7] Müller N (2018). Inflammation in schizophrenia: pathogenetic aspects and therapeutic considerations. Schizophr Bull.

[8] Khandaker GM, Cousins L, Deakin J, Lennox BR, Yolken R, Jones PB (2015). Inflammation and immunity in schizophrenia: implications for pathophysiology and treatment. Lancet Psychiatry.

[9] Williams JA, Burgess S, Suckling J, Lalousis PA, Batool F, Griffiths SL (2022). Inflammation and brain structure in schizophrenia and other neuropsychiatric disorders: a mendelian randomization study. JAMA Psychiat.

[10] Bethlehem RAI, Seidlitz J, White SR, Vogel JW, Anderson KM, Adamson C (2022). Brain charts for the human lifespan. Nature.

[11] van Erp TGM, Walton E, Hibar DP, Schmaal L, Jiang W, Glahn DC (2018). Cortical brain abnormalities in 4474 individuals with schizophrenia and 5098 control subjects via the Enhancing Neuro Imaging Genetics Through Meta Analysis (ENIGMA) Consortium. Biol Psychiatry.

[12] Di Sero A, Jørgensen KN, Nerland S, Melle I, Andreassen OA, Jovicich J (2019). Antipsychotic treatment and basal ganglia volumes: exploring the role of receptor occupancy, dosage and remission status. Schizophr Res.

[13] Lesh TA, Tanase C, Geib BR, Niendam TA, Yoon JH, Minzenberg MJ (2015). A multimodal analysis of antipsychotic effects on brain structure and function in first-episode schizophrenia. JAMA Psychiat.

[14] van Haren NE, Schnack HG, Cahn W, van den Heuvel MP, Lepage C, Collins L (2011). Changes in cortical thickness during the course of illness in schizophrenia. Arch Gen Psychiatry.

[15] Andreasen NC, Nopoulos P, Magnotta V, Pierson R, Ziebell S, Ho B-C (2011). Progressive brain change in schizophrenia: a prospective longitudinal study of first-episode schizophrenia. Biol Psychiatry.

[16] Yang C, Tang J, Liu N, Yao L, Xu M, Sun H (2021). The effects of antipsychotic treatment on the brain of patients with first-episode schizophrenia: a selective review of longitudinal MRI studies. Front Psychiatry.

[17] Grasby KL, Jahanshad N, Painter JN, Colodro-Conde L, Bralten J, Hibar DP (2020). The genetic architecture of the human cerebral cortex. Science.

[18] Cui L-B, Yin H (2022). The Xi’an Schizophrenia Imaging Lab (SIL) data and ten years of MRI study on schizophrenia. Psychoradiology.

[19] Stein DJ, Phillips KA, Bolton D, Fulford KW, Sadler JZ, Kendler KS (2010). What is a mental/psychiatric disorder? From DSM-IV to DSM-V. Psychol Med.

[20] Cui L-B, Liu L, Wang H-N, Wang L-X, Guo F, Xi Y-B (2018). Disease definition for schizophrenia by functional connectivity using radiomics strategy. Schizophr Bull.

[21] Cui L-B, Wei Y, Xi Y-B, Griffa A, De Lange SC, Kahn RS (2019). Connectome-based patterns of first-episode medication-naïve patients with schizophrenia. Schizophr Bull.

[22] Hoffman RE, Hawkins KA, Gueorguieva R, Boutros NN, Rachid F, Carroll K (2003). Transcranial magnetic stimulation of left temporoparietal cortex and medication-resistant auditory hallucinations. Arch Gen Psychiatry.

[23] Kay SR, Fiszbein A, Opler LA (1987). The Positive and Negative Syndrome Scale (PANSS) for schizophrenia. Schizophr Bull.

[24] Gong YX (1982). Wechsler Adult Intelligence Scale-Revised in China.

[25] Hawrylycz MJ, Lein ES, Guillozet-Bongaarts AL, Shen EH, Ng L, Miller JA (2012). An anatomically comprehensive atlas of the adult human brain transcriptome. Nature.

[26] Wei Y, de Lange SC, Scholtens LH, Watanabe K, Ardesch DJ, Jansen PR (2019). Genetic mapping and evolutionary analysis of human-expanded cognitive networks. Nat Commun.

[27] Desikan RS, Ségonne F, Fischl B, Quinn BT, Dickerson BC, Blacker D (2006). An automated labeling system for subdividing the human cerebral cortex on MRI scans into gyral based regions of interest. Neuroimage.

[28] Arnatkeviciute A, Fulcher BD, Fornito A (2019). A practical guide to linking brain-wide gene expression and neuroimaging data. Neuroimage.

[29] Nalls MA, Couper DJ, Tanaka T, van Rooij FJA, Chen M-H, Smith AV (2011). Multiple loci are associated with white blood cell phenotypes. PLoS Genet.

[30] Keller MF, Reiner AP, Okada Y, van Rooij FJA, Johnson AD, Chen M-H (2014). Trans-ethnic meta-analysis of white blood cell phenotypes. Hum Mol Genet.

[31] Yu H, Yan H, Wang L, Li J, Tan L, Deng W (2018). Five novel loci associated with antipsychotic treatment response in patients with schizophrenia: a genome-wide association study. Lancet Psychiatry.

[32] Lizano P, Lutz O, Xu Y, Rubin LH, Paskowitz L, Lee AM (2021). Multivariate relationships between peripheral inflammatory marker subtypes and cognitive and brain structural measures in psychosis. Mol Psychiatry.

[33] Laskaris L, Mancuso S, Shannon Weickert C, Zalesky A, Chana G, Wannan C (2021). Brain morphology is differentially impacted by peripheral cytokines in schizophrenia-spectrum disorder. Brain Behav Immun.

[34] Alexander-Bloch AF, Shou H, Liu S, Satterthwaite TD, Glahn DC, Shinohara RT (2018). On testing for spatial correspondence between maps of human brain structure and function. Neuroimage.

[35] Burt JB, Helmer M, Shinn M, Anticevic A, Murray JD (2020). Generative modeling of brain maps with spatial autocorrelation. Neuroimage.

[36] Jackson AJ, Miller BJ (2020). Meta-analysis of total and differential white blood cell counts in schizophrenia. Acta Psychiatr Scand.

[37] Mazza MG, Capellazzi M, Lucchi S, Tagliabue I, Rossetti A, Clerici M (2020). Monocyte count in schizophrenia and related disorders: a systematic review and meta-analysis. Acta Neuropsychiatr.

[38] Sacks D, Baxter B, Campbell BCV, Carpenter JS, Cognard C, Dippel D (2018). Multisociety consensus quality improvement revised consensus statement for endovascular therapy of acute ischemic stroke. Int J Stroke.

[39] Melbourne JK, Rosen C, Chase KA, Feiner B, Sharma RP (2021). Monocyte transcriptional profiling highlights a shift in immune signatures over the course of illness in schizophrenia. Front Psychiatry.

[40] Bishop JR, Zhang L, Lizano P (2022). Inflammation subtypes and translating inflammation-related genetic findings in schizophrenia and related psychoses: a perspective on pathways for treatment stratification and novel therapies. Harv Rev Psychiatry.

[41] Sealock JM, Lee YH, Moscati A, Venkatesh S, Voloudakis G, Straub P (2021). Use of the PsycheMERGE Network to investigate the association between depression polygenic scores and white blood cell count. JAMA Psychiat.

[42] Tourjman V, Kouassi É, Koué M-È, Rocchetti M, Fortin-Fournier S, Fusar-Poli P (2013). Antipsychotics’ effects on blood levels of cytokines in schizophrenia: a meta-analysis. Schizophr Res.

[43] Hashimoto N, Ito YM, Okada N, Yamamori H, Yasuda Y, Fujimoto M (2018). The effect of duration of illness and antipsychotics on subcortical volumes in schizophrenia: analysis of 778 subjects. Neuroimage Clin.

[44] Ho B-C, Andreasen NC, Ziebell S, Pierson R, Magnotta V (2011). Long-term antipsychotic treatment and brain volumes: a longitudinal study of first-episode schizophrenia. Arch Gen Psychiatry.

[45] Gutiérrez-Galve L, Chu EM, Leeson VC, Price G, Barnes TR, Joyce EM (2015). A longitudinal study of cortical changes and their cognitive correlates in patients followed up after first-episode psychosis. Psychol Med.

[46] Konopaske GT, Dorph-Petersen K-A, Pierri JN, Wu Q, Sampson AR, Lewis DA (2007). Effect of chronic exposure to antipsychotic medication on cell numbers in the parietal cortex of macaque monkeys. Neuropsychopharmacology.

[47] Xie Y, Guan M, Wang Z, Ma Z, Wang H, Fang P (2021). rTMS induces brain functional and structural alternations in schizophrenia patient with auditory verbal hallucination. Front Neurosci.

[48] Horacek J, Brunovsky M, Novak T, Skrdlantova L, Klirova M, Bubenikova-Valesova V (2007). Effect of low-frequency rTMS on electromagnetic tomography (LORETA) and regional brain metabolism (PET) in schizophrenia patients with auditory hallucinations. Neuropsychobiology.

[49] Jung WH, Kim JS, Jang JH, Choi J-S, Jung MH, Park J-Y (2011). Cortical thickness reduction in individuals at ultra-high-risk for psychosis. Schizophr Bull.

[50] Sydnor VJ, Larsen B, Bassett DS, Alexander-Bloch A, Fair DA, Liston C (2021). Neurodevelopment of the association cortices: patterns, mechanisms, and implications for psychopathology. Neuron.

