# Supplementary material for: Transcriptional level of inflammation markers associates with short-term brain structural changes in first-episode schizophrenia
Source: BMC Med. 2023 Jul 10;21:250. doi: 10.1186/s12916-023-02963-y (PMC10332052; doi:10.1186/s12916-023-02963-y)
Supplement: Supplementary file 1 — Additional file 1: Supplementary Methods. Exclusion criteria. rTMS treatment. Brain transcriptional data. Table S1. Genes of interest related to inflammation and antipsychotic treatment response. Table S2. Shapiro-Wilk and Shapiro-Francia normality tests for inflammation markers. Table S3. Antipsychotics received at baseline for patients with schizophrenia. Table S4. Significant group by time interaction, group main effects, and time main effects on regional measurements among patients who were medicated at baseline (FDR significant results in bold font). Table S5. Correlation between structural changes and gene expression of antipsychotic treatment response and inflammation among patients who were medicated at baseline. Fig. S1. Change on PANSS score at baseline (T0) and follow-up period (T1). Fig. S2. Transcriptional level of gene sets of interest. [file 12916_2023_2963_MOESM1_ESM.docx]

**Supplementary Methods**

**Exclusion criteria**

Exclusion criteria for patients included: (1) presence of another psychiatric disorder; (2) history of receiving antipsychotics (or more than two weeks of antipsychotic medication for the replication dataset), history of repetitive transcranial magnetic or current stimulation, or a history of behavioral treatment; (3) history of clinically significant neurological, neurosurgical or medical illnesses; (4) substance abuse within the prior 30 days or substance dependence within the prior 6 months; (5) pregnancy or any other MR imaging contraindications, e.g., cardiac pacemakers and other metallic implants. Exclusion criteria for healthy controls included: (1) presence of any psychotic syndrome; (2) history of receiving antipsychotics, repetitive transcranial magnetic stimulation, transcranial current stimulation, or behavioral treatment; (3) history of clinically significant neurological, neurosurgical or medical illnesses; (4) substance abuse within the prior 30 days or substance dependence within the prior 6 months; (5) pregnancy or MR imaging contraindications, e.g., cardiac pacemakers and other metallic implants.

**rTMS treatment**

Thirty-six out of 38 patients received rTMS treatment during hospitalization. Low frequency rTMS treatment (1 Hz) was provided throughout the whole hospitalization. It was given twice a day and each time lasted 15 minutes with a stimulation time per pulse 10 seconds, movement threshold 80%, interval time 5 seconds, and 60 repetitions. The stimulation site was targeted at the left TPJ area (22). The stimulus was provided by YRD CCY-I magnetic stimulator (YIRUIDE Inc., Wuhan, China). During the rTMS intervention, the type and dosage of antipsychotic drugs of the patients were determined by the psychiatrists; after discharge from hospital, antipsychotics remained unchanged, without other adjuvant treatment.

**Brain transcriptional data**

Brain transcriptional data from six human donors were downloaded from the Allen Human Brain Atlas (AHBA) (25) (http://human.brain-map.org/) and processed following the previous study (26). The six donors (five males, one female) had no history of neuropsychiatric or neuropathological disorders. For each donor brain, RNA was isolated and quantified with microarray in tissue samples covering the cortical, subcortical, brainstem and cerebellar regions. Expression data were normalized within each brain as well as between brains to minimize non-biological artefacts (for details refer to: <http://help.brain-map.org/display/humanbrain/Documentation).> In total, normalized expression levels of 20,734 genes represented by 58,692 probes were obtained. Gene symbols were re-annotated with the HUGO Gene Nomenclature Committee (HGNC) database (http://biomart.genenames.org/). For each sample, expression levels of the same gene were averaged across probes, and then normalized within each sample.

To assign tissue samples to brain regions, we first extracted the MNI coordinates of each sample in the left hemisphere from the AHBA website. Then we parcellated the MNI 152 template into 68 cortical regions (34 per hemisphere) defined as the Desikan-Killiany atlas (27) using the FreeSurfer software. We next mapped each sample to the nearest gray matter voxel based on the shortest Euclidean distance to all gray matter voxels in the MNI 152 template. A 2 mm distance threshold was set to avoid inaccurate assignment (28). Gene expression of the same cortical region was averaged, and resulted in a matrix of size of 34 × 20,734 (region × genes) for each donor. Gene expression level was finally averaged across the six donors and normalized to *z* score across all cortical regions per gene.

**Tables**

**Table S1** Genes of interest related to inflammation and antipsychotic treatment response

| **Traits** | **Study** | **Genes of interest** | | | | |
| --- | --- | --- | --- | --- | --- | --- |
| Antipsychotics | [Yu](https://pubmed.ncbi.nlm.nih.gov/?term=Yu+H&cauthor_id=29503163) et al. (2018) (31) | MEGF10  SLC1A1  PCDH7  CNTNAP5  TNIK | | | | |
| Inflammation |  | Leukocyte | Monocyte | Lymphocytes | Neutrophil | Basophil |
|  | Nalls et al. (2011) (29) | MUC21  HCG22  C6orf15  CDSN  PSORS1C1  PSORS1C2  CCHCR1  GSDMB  ORMDL3  GSDMA  PSMD3  CSF3  MED24  SNORD124  THRA  NR1D1 | ITGA4  CERKL  GATA2  LOC90246  C3orf27  RPN1  EDG2 | PSORS1C3  HCG27  HLA-C  HLA-B  EPS15L1  CALR3  C19orf44  CHERP | GSDMB  ORMDL3  GSDMA  PSMD3  CSF3  MED24  SNORD124  THRA  NR1D1 | LOC90246  C3orf27  RPN1 |
|  | Keller et al. (2014) (30) | DARC  CXCL2  PSMD3  CDK6  ARPC2  IL1F10  HLA-B | ITGA4  C3orf27  MHC |  | CXCL2  CDK6  CSF3  MED24  PSMD3  AK123889 |  |

**Table S2** Shapiro-Wilk and Shapiro-Francia normality tests for inflammation markers

|  | Leukocyte (10⁹/L) | Neutrophils % | Lymphocyte % | Monocyte  % | Eosinophils % | Basophils  % |
| --- | --- | --- | --- | --- | --- | --- |
| *p*-value | 3.44E-06 | 0.01 | 0.16 | 0.69 | 5.51E-04 | 6.83E-11 |
| S-W Statistic | 0.73 | 0.92 | 0.96 | 0.98 | 0.86 | 0.23 |

**Table S3** Antipsychotics received at baseline for patients with schizophrenia

|  | Days | Dose (olanzapine equivalent, mg/day)^*^ |
| --- | --- | --- |
| Medicated patients (n = 31) | 5.78 ± 3.82 | 9.23 ± 4.67 |
| Unmedicated patients (n = 7) | / | / |

*Not available for 5 patients.

**Table S4** Significant group by time interaction, group main effects, and time main effects on regional measurements among patients who were medicated at baseline (FDR significant results in bold font)

|  | Group | | Time | | Group*Time | |
| --- | --- | --- | --- | --- | --- | --- |
|  | *t* value | *p* value | *t* value | *p* value | *t* value | *p* value |
| Subcortical regions | | | | | | |
| Right Pallidum | 1.03 | 0.31 | -1.08 | 0.28 | **2.65** | **0.01** |
| Left Pallidum | -0.01 | 0.99 | -1.88 | 0.06 | **2.96** | **<0.01** |
| Regional cortical thickness | | | | | | |
| Right entorhinal cortex | **3.66** | **<0.01** | -1.24 | 0.22 | 0.44 | 0.66 |
| Right superior frontal gyrus | **-4.97** | **<0.01** | 0.88 | 0.38 | **-3.23** | **<0.01** |
| Left rostral middle frontal gyrus | **-2.84** | **0.01** | **3.75** | **<0.01** | **-3.53** | **<0.01** |
| Left caudal anterior cingulate gyrus | 0.63 | 0.53 | 1.63 | 0.11 | **-4.47** | **<0.01** |
| Left superior frontal gyrus | **-3.20** | **<0.01** | 0.44 | 0.66 | **-4.37** | **<0.01** |
| Left superior parietal lobule | -0.91 | 0.37 | -0.60 | 0.55 | **2.70** | **0.01** |
| Right inferior parietal lobule | -1.27 | 0.21 | -1.76 | 0.08 | **3.15** | **<0.01** |
| Right lateral occipital lobule | -0.33 | 0.74 | -1.44 | 0.15 | **3.67** | **<0.01** |
| Right superior parietal lobule | 0.06 | 0.95 | -1.41 | 0.16 | **3.18** | **<0.01** |

**Table S5** Correlation between structural changes and gene expression of antipsychotic treatment response and inflammation among patients who were medicated at baseline

| Neuroimaging | Study | Gene set of interests | Pearson r | Pearson p |
| --- | --- | --- | --- | --- |
| Cortical thickness change | Yu et al. (2018) | Antipsychotics | 0.243 | 0.166 |
|  | Nalls et al. (2011) | Leukocyte | 0.061 | 0.731 |
|  |  | Neutrophil | 0.199 | 0.259 |
|  |  | Basophil | 0.423 | 0.013 |
|  |  | Monocyte | **0.642** | **<0.001** |
|  |  | Lymphocytes | 0.147 | 0.407 |
|  | Keller et al. (2014) | Leukocyte | -0.168 | 0.341 |
|  |  | Neutrophil | -0.050 | 0.778 |
|  |  | Monocyte | **0.552** | **0.001** |
| Surface area change | Yu et al. (2018) | Antipsychotics | 0.361 | 0.036 |
|  | Nalls et al. (2011) | Leukocyte | 0.050 | 0.780 |
|  |  | Neutrophil | 0.048 | 0.788 |
|  |  | Basophil | -0.196 | 0.267 |
|  |  | Monocyte | -0.051 | 0.774 |
|  |  | Lymphocytes | 0.332 | 0.055 |
|  | Keller et al. (2014) | Leukocyte | 0.208 | 0.237 |
|  |  | Neutrophil | -0.100 | 0.574 |
|  |  | Monocyte | 0.088 | 0.623 |

**Figures**


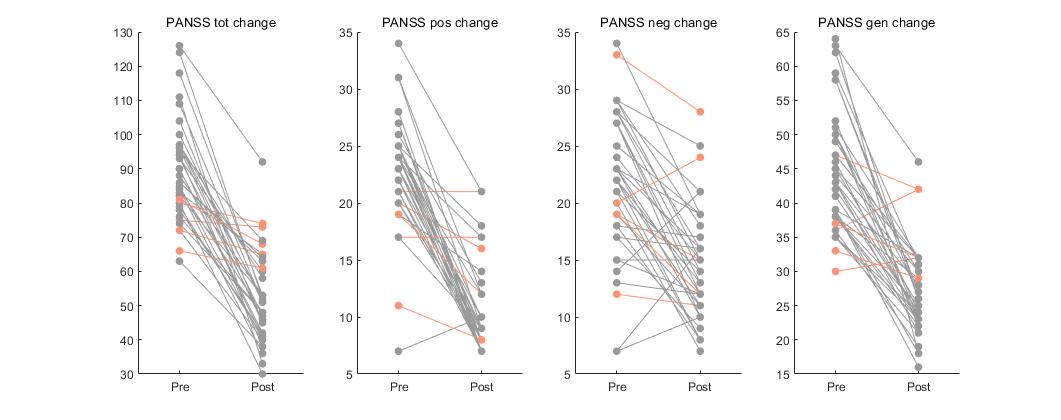


**Figure S1**. Change on PANSS score at baseline (T_0_) and follow-up period (T_1_). PANSS change rate was calculated as (PANSS_T_1_−PANSS_T_0_)/(PANSS_T_0_−30). Patients with a significant symptom reduction on PANSS total score (over 30%) were plotted as gray points, others were plotted in orange color.

**
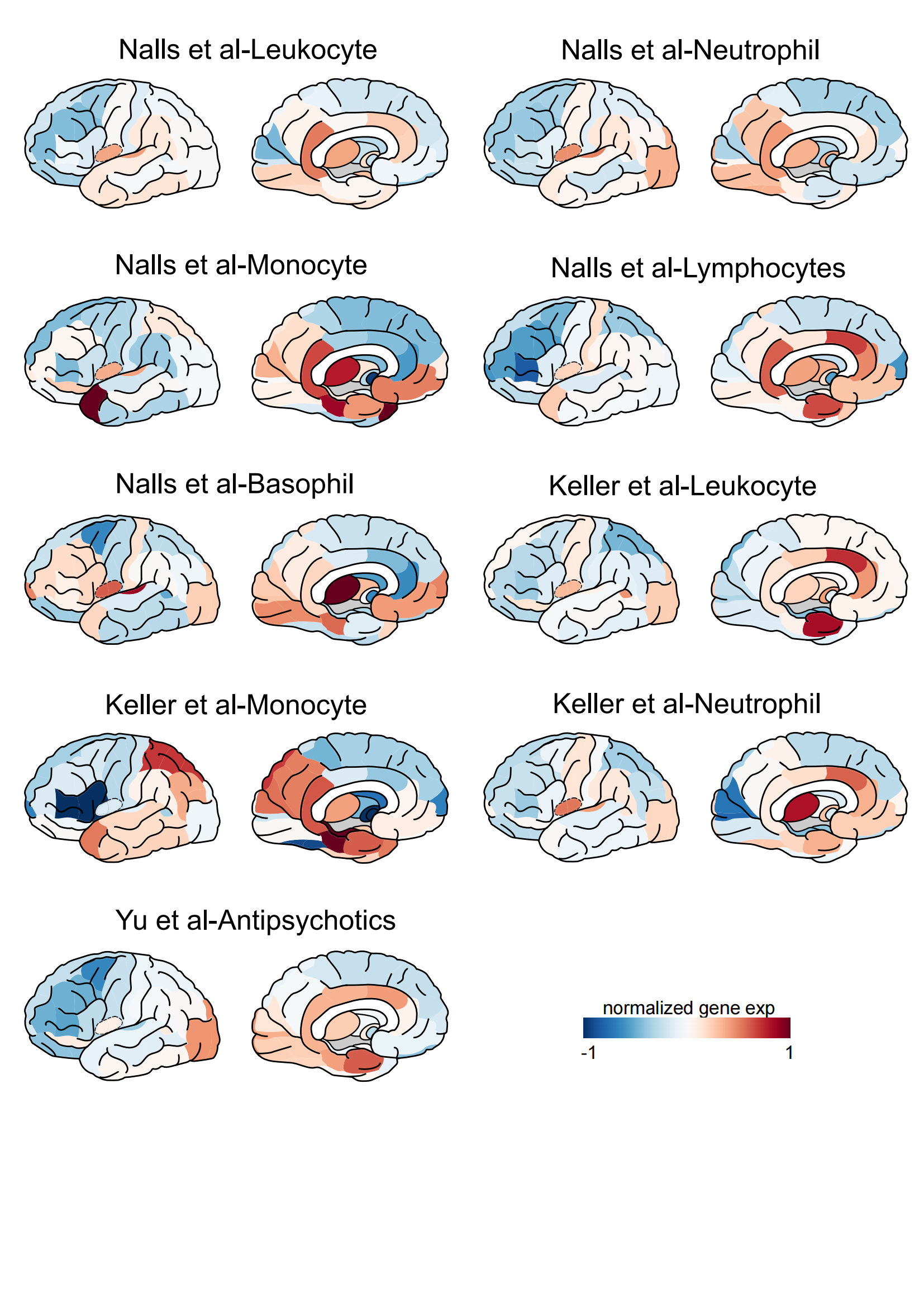
**

**Figure S2**. Transcriptional level of gene sets of interest. Genes related to inflammation (Nalls et al. (29), Keller et al. (30)) and antipsychotic treatment response (Yu et al. (31)) were listed in Table S1. Brain transcriptional data were obtained from the Allen Human Brain Atlas (AHBA). Gene expression level was averaged across the six donors and normalized to z score across all cortical regions.
